# Tamoxifen induces oxidative stress and apoptosis in oestrogen receptor-negative human cancer cell lines

**DOI:** 10.1038/sj.bjc.6690042

**Published:** 1999-01

**Authors:** C Ferlini, G Scambia, M Marone, M Distefano, C Gaggini, G Ferrandina, A Fattorossi, G Isola, P Benedetti Panici, S Mancuso

**Affiliations:** Laboratory of Antineoplastic Pharmacology, Zeneca, and Department of Obstetrics and Gynecology, Catholic University of Sacred Heart, Rome, Italy

**Keywords:** oxidative stress, tamoxifen, apoptosis, NF-κB

## Abstract

Recent data have demonstrated that the anti-oestrogen tamoxifen (TAM) is able to facilitate apoptosis in cancer cells not expressing oestrogen receptor (ER). In an attempt to identify the biochemical pathway for this phenomenon, we investigated the role of TAM as an oxidative stress agent. In two ER-negative human cancer cell lines, namely T-leukaemic Jurkat and ovarian A2780 cancer cells, we have demonstrated that TAM is able to generate oxidative stress, thereby causing thiol depletion and activation of the transcriptional factor NF-κB. As described for other oxidative agents, TAM was able to induce either cell proliferation or apoptosis depending on the dose. When used at the lowest dose tested (0.1 μM), a slight proliferative effect of TAM was noticed in terms of cell counts and DNA synthesis rate, whereas at higher doses (10 μM) a consistent occurrence of apoptosis was detected. Importantly, the induction of apoptosis by TAM is not linked to down-regulation or functional inactivation by phosphorylation of the antiapoptotic bcl-2 protein. © 1999 Cancer Research Campaign


*British Journal of Cancer* (1999) **79**(2), 257–263
© 1999 Cancer Research Campaign

### Tamoxifen induces oxidative stress and apoptosis in oestrogen receptor-negative human cancer cell lines

**C Ferlini, G Scambia, M Marone, M Distefano, C Gaggini, G Ferrandina, A Fattorossi, G Isola, P Benedetti Panici and S Mancuso**

Laboratory of Antineoplastic Pharmacology, Zeneca, and Department of Obstetrics and Gynecology, Catholic University of Sacred Heart, Rome, Italy

**Summary** Recent data have demonstrated that the anti-oestrogen tamoxifen (TAM) is able to facilitate apoptosis in cancer cells not expressing oestrogen receptor (ER). In an attempt to identify the biochemical pathway for this phenomenon, we investigated the role of TAM as an oxidative stress agent. In two ER-negative human cancer cell lines, namely T-leukaemic Jurkat and ovarian A2780 cancer cells, we have demonstrated that TAM is able to generate oxidative stress, thereby causing thiol depletion and activation of the transcriptional factor NF-κB. As described for other oxidative agents, TAM was able to induce either cell proliferation or apoptosis depending on the dose. When used at the lowest dose tested (0.1 μM), a slight proliferative effect of TAM was noticed in terms of cell counts and DNA synthesis rate, whereas at higher doses (10 μM) a consistent occurrence of apoptosis was detected. Importantly, the induction of apoptosis by TAM is not linked to down-regulation or functional inactivation by phosphorylation of the antiapoptotic bcl-2 protein.

**Keywords:** oxidative stress; tamoxifen; apoptosis; NF-κB

Although several types of programmed cell death (PCD) have been characterized, apoptosis is considered the predominant mode of PCD that occurs under physiological conditions. The relevance of apoptosis in oncology appears evident when considering that oncogenic transformation can be determined by a defect in apoptosis. Moreover, one of the aims of anti-tumour chemotherapy is to obtain tumour regression by stimulating apoptosis in cancer cells as specifically as possible. One possible way to induce apoptosis in cancer cells is by blocking signal transduction of growth factors and hormone receptors. In fact, some tumour cells, such as in the prostate and breast cancers, express receptors for endogenous hormones, and these findings have led to the development and clinical use in cancer therapy of molecules endowed with anti-hormone properties. The anti-oestrogen tamoxifen (TAM) was the first drug clinically developed in this class of compounds and has been widely and successfully utilized in the treatment of breast cancer. For this disease, large clinical trials have indicated that a favourable response to adjuvant therapy with this drug is present also in oestrogen receptor (ER)-poor tumours (reviewed in Jaiyesimi et al, 1995), thereby suggesting that at least a part of the antineoplastic action may not be related to the anti-hormone properties of TAM. This hypothesis gains support from early data of Perry et al (1995) showing that at a clinically achievable dosage, TAM reduces the growth and induces apoptosis in ER-negative cell lines. More recently, our group has demonstrated in an ER-negative model that TAM facilitates apoptosis induced by docetaxel (Ferlini et al, 1997), whose apoptotic activity is dependent on the stabilization of the microtubule network and inactivation via phosphorylation of the antiapoptotic bcl-2 protein (Haldar et al, 1996).

*Received 24 July 1997*
*Revised 6 May 1998*
*Accepted 8 May 1998*

*Correspondance to:* S Mancuso, Department of Obstetrics and Gynecology, Catholic University of Sacred Heart, L. go Agostino Gemelli 8, 00168 Rome, Italy

In principle, several in vitro properties of TAM could be held responsible for its pro-apoptotic role in ER-negative cells: calcium channel blocking activity (Lopes et al, 1990), inhibition of protein kinase C (Issandou et al, 1990), up-regulation of c-*myc* expression (Kang et al, 1996) or induction of oxidative stress (Gundimeda et al, 1996; Ye et al, 1996). In particular, this last point is intriguing as previous studies have indicated TAM to be an antioxidant agent (Wiseman et al, 1990, 1993).

The aim of the present work was to identify an ER-independent pathway that could explain, at least in part, the antineoplastic activity of TAM reported in ER-negative cells and ER-poor tumours. The results indicate that TAM acts as a potent pro-oxidative agent able to induce activation of the nuclear transcription factor kappa B (NF-κB), thereby establishing a theoretical ground to design new drug approaches combining TAM with chemotherapy.

### MATERIALS AND METHODS

### Drugs

TAM stock solutions (10 mM) were made in absolute ethanol, and were used at concentrations ranging from 0.1 to 10 μM. The final ethanol concentration never exceeded 1% (v/v), in control and treated samples.

### Cell cultures

The cell lines used in this study were the ovarian cancer A2780 cells and the T-leukaemic Jurkat. For all the cell lines, RPMI-1640 was used as culture medium and supplemented with 10% fetal calf serum (FCS) and 200 U $ml^{-1}$ penicillin. A2780 adherent cells, propagated as monolayer culture in 75-$cm^2$ tissue-culture flasks, were trypsinized weekly and plated at a density of $8 \times 10^4$ cells $ml^{-1}$.

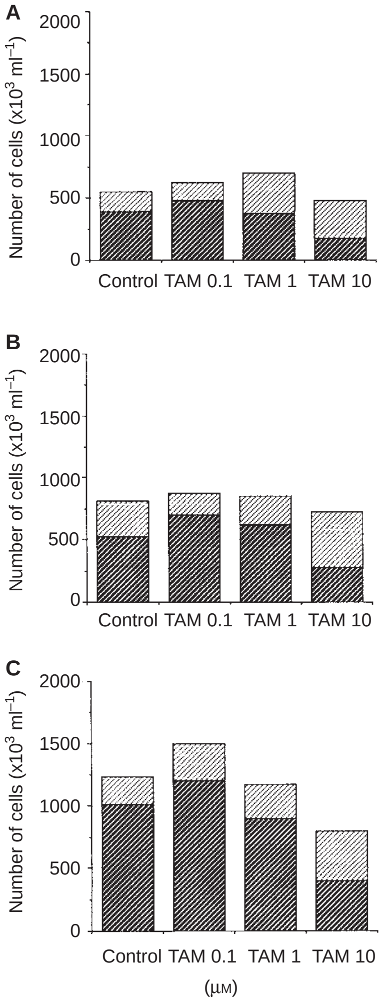

**Figure 1** Cell count analysis performed in Jurkat cells after (**A**) 24, (**B**) 48, and (**C**) 72 h of culture in the presence of TAM (concentration range 0.1–10 μM). Viable cells (▨) and apoptotic cells (▨) were counted in parallel preparations. Standard deviation was less than 5% for each count and has been omitted

**Table 1** Cell cycle analysis of Jurkat cells cultured in the presence of TAM

| | Time of exposure (h) | $G_1$ | S | $G_2$ | DNA fragmentation[a] |
|---|---|---|---|---|---|
| Control | 6 | 55.8 | 37.7 | 6.5 | 8.64 |
| TAM 0.1 μM | | 55.3 | 39 | 5.7 | 6.98 |
| TAM 1 μM | | 54.6 | 39 | 6.4 | 5.91 |
| TAM 10 μM | | 52.3 | 40.2 | 7.5 | 6.89 |
| Control | 24 | 76.1 | 16.2 | 7.7 | 9.13 |
| TAM 0.1 μM | | 75.9 | 16.2 | 7.9 | 8.87 |
| TAM 1 μM | | 76.4 | 16.3 | 7.3 | 9.28 |
| TAM 10 μM | | 77.3 | 12.9 | 9.8 | 16.98 |
| Control | 48 | 59.3 | 28.5 | 12.2 | 7.82 |
| TAM 0.1 μM | | 60.1 | 28.2 | 11.7 | 7.67 |
| TAM 1 μM | | 61 | 26.6 | 12.5 | 6.55 |
| TAM 10 μM | | 75.2 | 14.4 | 10.3 | 26.19 |
| Control | 72 | 54.9 | 32.8 | 12.3 | 3.12 |
| TAM 0.1 μM | | 55.1 | 32.6 | 12.3 | 3.83 |
| TAM 1 μM | | 54.5 | 33.7 | 11.8 | 3.34 |
| TAM 10 μM | | 71.7 | 17.8 | 10.5 | 27.66 |

[a]DNA fragmentation was excluded by cell cycle analysis.

Jurkat cells (growing in suspension) were seeded at 2–3×10$^5$ cells ml$^{-1}$ and split in a ratio of 1:3 every day. All cultures were incubated at 37°C under 5% carbon dioxide/95% air in a high humidity atmosphere. Both cell lines used were ER negative (Berman et al, 1991).

### Growth experiments

Cells were plated in six-well flat-bottom plates (Falcon, Lincoln Park, NJ, USA) at a concentration of 1×10$^5$ cells ml$^{-1}$ in complete medium. After 24 h, the medium was replaced with fresh medium containing TAM and incubation was continued up to 72 h. Control cells were treated with vehicle alone. Quadruplicate counts of triplicate cultures were performed at each time point (24, 48 and 72 h).

### [$^3$H]Thymidine uptake

Jurkat cells were plated in 96-well flat-bottom plates (Costar, Cambridge, MO, USA) at a concentration of 1×10$^5$ cells ml$^{-1}$ in 200 μl of complete medium containing TAM and incubated from 6 to 72 h. Control cells were treated with vehicle alone. In the last 6 h of culture, 0.5 μCi per well of [$^3$H]thymidine (Amersham Italia, Milan, Italy) was added. Plates were harvested and counted using the Betaplate system (Wallac Oy, Turku, Finland). Results are expressed as c.p.m. means of six replicate cultures. Experimental data was considered acceptable if the standard deviation was less than 15% of the c.p.m. mean calculated using six replicate samples.

### Fluorescent probes and flow cytometry

The redox state was investigated using 5-chloromethylfluorescein diacetate (CMFDA, Molecular Probes, Eugene, OR, USA) and 2-7-dichlorodihydrofluorescein diacetate (DCHFDA, Molecular Probes). Cells were incubated at 37°C with 10 nM CMFDA used as an indicator of intracellular thiol levels (Poot et al, 1991). After 20 min, samples were washed once and then resuspended in phosphate-buffered saline (PBS) containing 4 μg ml$^{-1}$ ethidium bromide (EB), a probe for plasma membrane status. A parallel culture was stained at 37°C in complete medium with 5 μM DCHFDA, an indicator of reactive oxygen species (ROS) production (Bass et al, 1983). After 1 h of incubation, cells were washed once and then resuspended in PBS containing 5 μM DCHFDA and 4 μg ml$^{-1}$ EB. Dual-colour flow cytometry was performed by collecting the fluorescence of DCHFDA and CMFDA in the green and that of EB in the orange emissions. Electronic colour compensation was used to compensate for crossover between fluorochromes. Fluorescence of EB served to gate out dead cells. Up to 15 000 events were acquired using Lysis II software (Becton Dickinson Immunocytometry Systems, San Jose, CA, USA) on a FACScan (Becton Dickinson) cytometer equipped with a standard filter set for green (530/30 nm) and orange emissions (585/42 nm). Fluorescence signals were acquired in log mode. Results are expressed as the mean channel of fluorescence intensity.

### Cell cycle analysis

Cells were plated in the specific medium supplemented as above. After 24 h, the medium was replaced with fresh medium containing TAM or vehicle alone. After various times of culture (from 6 h to 72 h), cells were harvested and nuclei isolated and

 © Cancer Research Campaign 1999

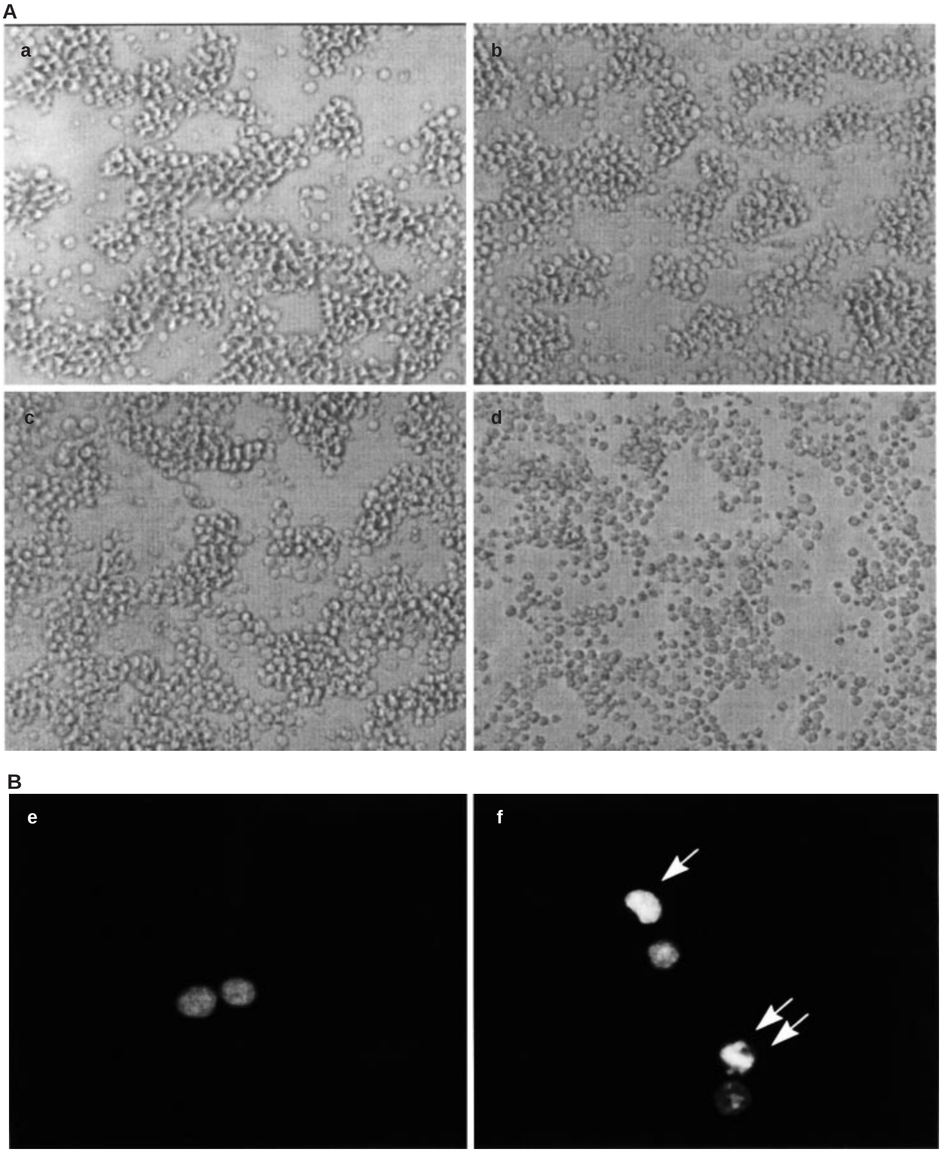

**Figure 2** Representative morphological analysis without (**A**) or with (**B**) DNA staining of Jurkat cells after 72 h of culture in the presence of TAM (concentration range 0.1–10 μM). (**A**) (magnification 100×) control cells are characterized by growth in clumps forming a large-meshed net on the plate. At the lowest TAM dose (0.1 μM), clumps appear slightly larger as an indication of the increased proliferative activity. At the highest TAM dose, cell clumping is no longer visible as proliferative activity is severely impaired. (**a**) Control; (**b**) TAM 0.1 μM; (**C**) TAM 1 μM; (**D**) TAM 10 μM. (**B**) (magnification 1000×) features of nuclear apoptosis with extremely condensed and polarized chromatin (arrows) and nuclear blebbing (double arrows). (**e**) Control; (**f**) TAM 10 μM

stained using a solution containing 0.1% (w/v) sodium citrate, 0.1% (v/v) Nonidet-P40, 4 mM EDTA and 50 μg $ml^{-1}$ of propidium iodide (PI) as a DNA dye (Ferlini et al, 1996). Incubation of the cells with the staining solution lasted a minimum of 24 h at 4°C.

Flow cytometric DNA analysis was performed by acquiring a minimum of 20 000 nuclei. DNA fluorescence was collected in linear and log mode and pulse signal processing was used to set a doublet discrimination gate. Cell cycle analysis was performed using the Multicyle software package (Phoenix, San Diego, CA, USA).

### Electrophoretic mobility shift assay (EMSA)

About $20 \times 10^6$ cells were resuspended in 200 μl high-salt buffer (20 mM Hepes, pH 7.9, 400 mM sodium chloride, 1 mM EDTA, 1 mM EGTA, 1 mM DTT, 1 mM PMSF, 0.6% Nonidet-P40). After a 20 min incubation on ice, the samples were centrifuged and the supernatant was stored in aliquots at –70°C. The protein concentration was determined with the Bio-Rad Protein Assay (BioRad Laboratories, Hercules, CA, USA). Equal amounts of the extracts (15 μg) were added to 6 μg of poly(dI–dC) as a non-specific DNA competitor in 1× binding buffer (10 mM Hepes, pH 7.5, 60 mM potassium chloride, 1 mM EDTA, 1 mM DTT, 10% glycerol). After a 15 min incubation on ice, 20 000 c.p.m. of the NF-κB or AP-1 specific probes were added and incubated with the reaction mixture for 20 min at room temperature. Samples were loaded on a 6% polyacrylamide gel and run in 0.3 × TBE buffer at 150 V. Gels were exposed in an Instant Imager Electronic Autoradiography Instrument (Packard Instrument, Meriden, CT, USA) and were quantified by the Imager program provided with the instrument. The sequences of the oligonucleotides were as described in Los et al (1995).

© Cancer Research Campaign 1999

### Bcl-2 Western blots

The pellet obtained from $10 \times 10^6$ cells was washed twice in 1× PBS and then dissolved in lysis buffer containing 20 mM tris-HCl, pH 7.4, 100 mM sodium chloride, 5 mM magnesium chloride, 1% Nonidet P-40, 0.5% sodium deoxycholate, 2 U ml$^{-1}$ of the kallikrein inhibitor aprotinin. After addition of sodium dodecyl sulphate (SDS) sample buffer, 200 μg total proteins were separated by 15% sodium dodecyl sulphate polyacrylamide gel electrophoresis (SDS-PAGE) and electroblotted onto PVDF (Millipore, Bedford, MA, USA). The membranes were incubated with 6% non-fat dry milk in 1× TBST (0.1 M trizma base, 0.15 M sodium chloride, 0.05% Tween 20, pH 7.4) for blocking and then with a 1:100 dilution of the mouse monoclonal anti-Bcl-2 antibody clone 124 (Dako, Glostrup, Denmark) in 1× TBST. The membranes were then incubated with a biotinylated secondary antibody (ABC Vectastain Elite, Vector Labs, Burlingame, CA, USA) and detection was performed with the DAB kit (Vector Labs) in 1× TBST. Images of the blots were acquired with a CCD camera and quantification of the Bcl-2 bands were performed by Phoretix 1D Gel Analysis software (Phoretix International, Newcastle upon Tyne, UK).

### Morphological analysis

Morphological features of apoptosis were assessed by scoring control and TAM-treated cultures (concentration range 0.1–10 μM) seeded in six-well flat bottom plates (Costar) using an inverted Diavert fluorescence microscope (Leica, Wetzlar, Germany). Cultures were fixed in 70% cold ethanol, stored overnight at –20°C, resuspended in PBS containing 50 μg ml$^{-1}$ ribonuclease (Sigma) and stained with the green DNA dye Yoyo-1 (Molecular Probes) at a concentration of 4 μM. Samples were then examined to identify cell with features of apoptotic chromatin as previously described (Ferlini et al, 1996). Image analysis was performed using the IAS2000 system (Delta Sistemi, Rome, Italy).

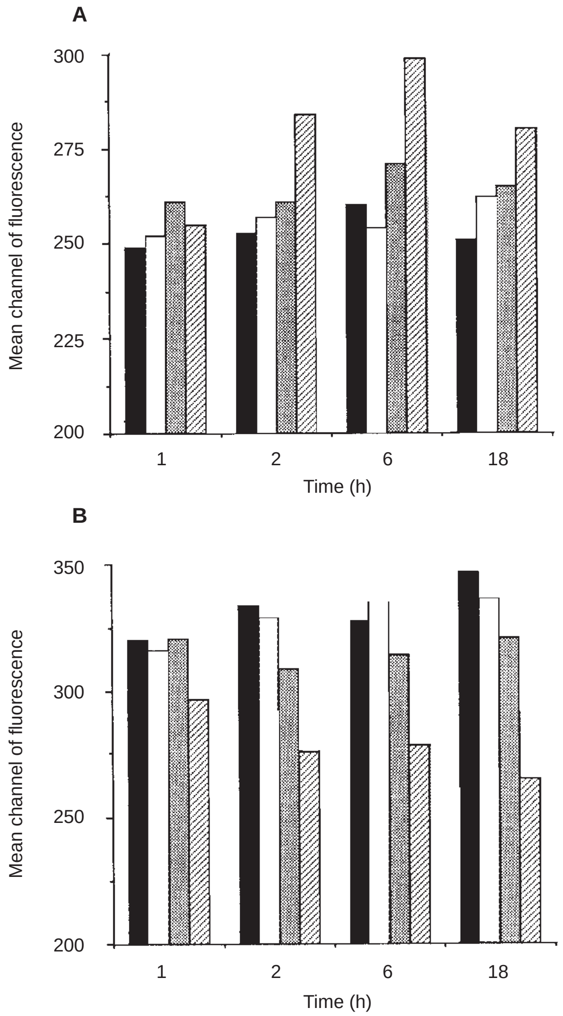

**Figure 3** Flow cytometric analysis of redox metabolism in Jurkat cells after 1, 2, 6 and 18 h of culture in the presence of 0.1 μM (▩), 1 μM (▨) and 10 μM (□) of TAM. Control cells were treated with vehicle alone (■). ROS production (**A**) and thiol level (**B**) were monitored using the fluorescent probes DCHFDA and CMFDA respectively. The same experiment was repeated three times with similar results

### RESULTS

### ER-independent induction of proliferation/apoptosis

Jurkat cells were cultured in the presence of TAM (concentration range 0.1–10 μM) for up to 72 h. After 24, 48 and 72 h, cell count analysis demonstrated (Figure 1) a slight proliferative effect at the lowest TAM dose (0.1 μM). In contrast, a decrease in live cells occurred when TAM was used at 10 μM (Figure 1). To evaluate whether the observed effect was linked to a perturbation in cell cycle, DNA content analysis was performed after 6, 24, 48 and 72 h (Table 1). Results showed that an appreciable cytokinetic effect was present only in the presence of 10 μM TAM and that a cell cycle arrest in the Gl/S boundary was evident starting from 8 h. A concomitant increase of DNA fragmentation, suggestive for the occurrence of late apoptosis, was also evident (Table 1). To confirm the presence of apoptosis, morphological analysis was performed and cells with features of apoptotic chromatin, such as condensation and blebbing (Figure 2), were counted after staining with the green DNA dye Yoyo-1. Results confirmed (Figure 1) that a stable (during time course) increase of apoptotic cells was present at the highest concentration of TAM (10 μM). Remarkably, it is worth noting that at this dosage a reliable antiproliferative effect was not visible until 72 h because the sum of apoptotic and live cells in TAM-treated samples did not detectably decrease with respect to control cells after 24 and 48 h of culture.

In order to assess DNA synthesis rate in Jurkat cells cultured in the presence of TAM (concentration range 0.1–10 μM), [$^3$H]-thymidine uptake was evaluated after 6, 24, 48 and 72 h of culture (data not shown). Up to 24 h, no significant changes were evident in all culture conditions. Conversely, after 48 and 72 h a slight increase in [$^3$H]thymidine uptake was noticed for 0.1 μM of TAM, whereas at 10 μM [$^3$H]thymidine uptake decreased remarkably.

Taking all these data together, we conclude that TAM is able to induce either cell proliferation or apoptosis depending on the dose, thereby resembling other mediators of oxidative stress (Murrell et al, 1990; Rao et al, 1992; Los et al, 1995).

### Redox metabolism in TAM-treated cells

As a direct approach to the question of whether TAM is able to modulate redox metabolism, ROS production and intracellular thiol level were evaluated using flow cytometry in two human cancer cells: the T-leukaemic Jurkat and the ovarian cancer A2780 cells. Such analysis was performed after 1, 2, 6 and 18 h of culture.

 *© Cancer Research Campaign 1999*

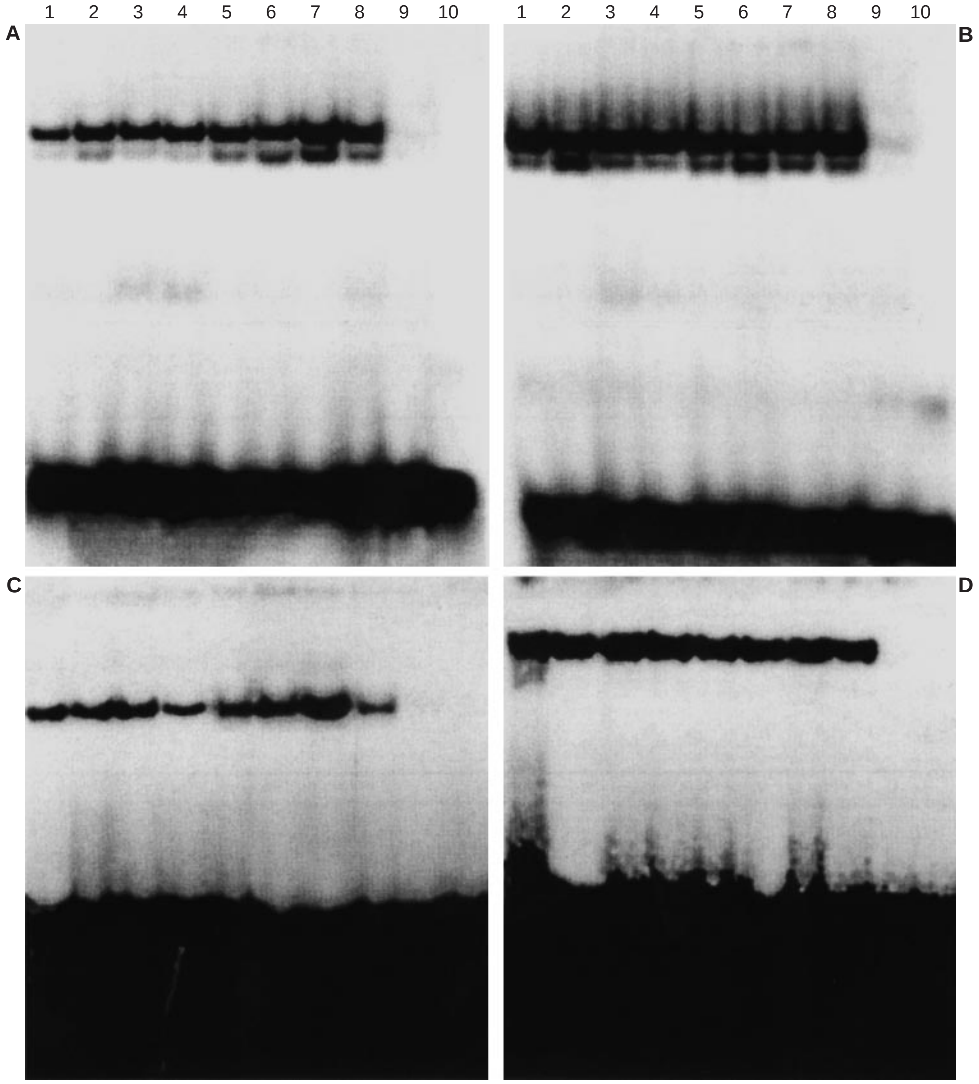

**Figure 4** DNA-binding activity of the transcription factors NF-kB (**A** and **C**) and AP-1 (**B** and **D**) in A2780 (**A** and **B**) and Jurkat cells (**C** and **D**). Lanes 1–4 represent control cells (only vehicle ethanol 1%) after 1, 2, 3 and 4 h of culture respectively. Lanes 5–8 are TAM-treated cells (10 μM) after 1, 2, 3 and 4 h of culture. Specificity of DNA binding was verified using a ten- and 100-fold excess of unlabelled oligonucleotide (lanes 9 and 10 respectively)

In Jurkat cells, an increased ROS production was noticed starting from 2 h of culture at the highest dose of TAM (10 μM) (Figure 3A). Remarkably, such increase in ROS production was combined with a decreased thiol level that was present at the same TAM concentration starting from 1 h (Figure 3B). Similar effects (increased ROS production and decreased thiol level), even if less evident, were present also at the lower TAM concentrations (0.1 and 1 μM). Similar results were obtained using A2780 cells (not shown). This suggests that TAM was able to behave as a pro-oxidative agent, sequentially inducing thiol consumption and oxidative stress in a dose-dependent manner.

### Modulation of transcription factor activity induced by TAM

At least two well-defined transcription factors, NF-κB and AP-1, are regulated by intracellular redox status (Sen et al, 1996). Thus, we evaluated the DNA-binding activity of these two transcription factors using EMSA in both ER-negative cancer cell lines upon a short exposure (range 1–4 h) to 10 μM of TAM, the dose inducing maximal oxidative stress and apoptosis. In Jurkat cells, an increased DNA-binding activity was documented for NF-κB, whereas the activity of AP-1 remained essentially unaffected (Figure 4). The DNA-binding activity of NF-κB peaked in Jurkat cells after 3 h of TAM treatment with a maximum increase up to 3.7-fold compared with the control value. Similarly, A2780 cells showed an increase of the DNA-binding activity of NF-κB up to 3.2-fold after 3 h of culture in the presence of TAM (Figure 4), without an appreciable change of DNA binding activity of AP-1.

### Bcl-2 expression and phosphorylation

To better clarify the biochemical mechanism underlying TAM-induced apoptosis, we looked at the expression and activation status of the antiapoptotic bcl-2 protein. Jurkat cells were treated either with the vehicle alone (ethanol 1%) or with 10 μM TAM for 6, 24, 48 and 72 h. An additional control was carried out using Jurkat cells cultured in the presence of 1 μM paclitaxel for 24 h to

© Cancer Research Campaign 1999

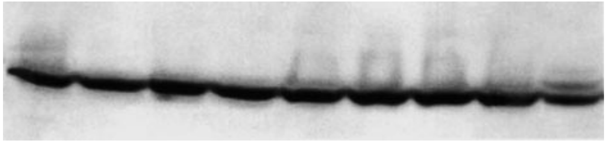

**Figure 5** Western blots of Bcl-2 in Jurkat cells. Lanes 1–4 are control cells after 6, 24, 48 and 72 h of culture respectively. Lane 5–8 are TAM-treated cultures (10 µM) after 6, 24, 48 and 72 h of culture. Lanes 9 is the positive control obtained after 24 h of culture in the presence of 1 µM of paclitaxel, showing the slow-migrating phosphorylated and hyperphosphorylated isoforms of bcl-2

induce the phosphorylated slow isoform of bcl-2 protein. Results clearly indicated that TAM was unable to down-regulate the expression of bcl-2 protein or to induce its functional inactivation by phosphorylation (Figure 5), thereby suggesting for TAM that oxidative stress, thiol depletion and activation of NF-κB are not involved in the regulation of bcl-2 activity.

### DISCUSSION

Large clinical trials have demonstrated the efficacy of the anti-oestrogen TAM in the adjuvant therapy of breast cancer. Although the effect of TAM in terms of recurrence-free survival is maximum in ER-rich tumours, a significant favourable effect was detected also in ER-poor cancers (reviewed in Jaiyesimi et al, 1995). Interestingly, responses to TAM have been observed also in cancers not derived from oestrogen-sensitive tissues, such as glioma (Couldwell et al, 1994) and melanoma (McClay et al, 1993). Perry et al (1995) proposed a possible explanation for these data by reporting that TAM is able to induce apoptosis via an ER-independent pathway. In this paper, we furnish a biochemical basis for the TAM-induced apoptosis in ER-negative models, by demonstrating that TAM generates ROS production and thiol depletion in a dose-dependent manner. The balance between ROS and cellular thiol levels plays a pivotal role in regulating cell cycle progression and apoptosis. It has been previously shown that mitogenic stimulation of cells induces a rapid and transient increase of ROS production (Murrell et al, 1990; Rao et al, 1992; Los et al, 1995), suggesting that a short pro-oxidative shift provides a signal to enter the cell cycle. This oxidative burst, with its consequent gene expression, must be balanced and counteracted by endogenous antioxidants. In keeping with this view, TAM at 0.1 µM stimulates, in a slight but consistent way, DNA synthesis and cell proliferation as indicated by cell counts and [$^3$H]thymidine uptake. Conversely, increasing the dose of TAM leads to opposite effects along the same pathway. In fact, at 10 µM of TAM, cell proliferation is not immediately blocked, rather a higher number of cells enter into the apoptotic pathway after an initial progression into the proliferative cycle perhaps as a consequence of excessive ROS production and a non-sustainable thiol depletion. Only later, in surviving cells, the proliferative compartment is clearly reduced and the cell cycle is frozen at the Gl/S boundary.

As in other models of oxidative stress, TAM treatment increases ROS until a threshold level is attained for the activation of NF-κB, which then translocates into the cell nucleus thereby promoting transcription of a package of dependent genes. Among these, NF-κB contributes to the control of c-*myc* activity, an oncogene essential for the cell proliferation/apoptosis programme (Janssen et al, 1995). In this scenario, our data are in good agreement with Kang et al (1996), who reported that TAM regulates c-*myc* expression at the transcriptional level: low enhancement of c-*myc* results in the cell cycle progression, whereas higher enhancement led to cytostasis and apoptosis. Thus, it appears that oxidative stress, NF-κB activation, c-*myc* expression and the proliferation/apoptosis programme are sequentially involved in the same pathway by TAM treatment.

In contrast to the oxidative agent okadaic acid (Haldar et al, 1995), ROS production upon TAM treatment is unable to produce the serine-phosphorylated isoform of bcl-2 protein that, in this inactivated form, can no longer prevent lipid peroxidation and apoptosis (Hockenbery et al, 1993). Moreover, a down-regulation of the bcl-2 protein does not appear to be involved in the induction of apoptosis upon TAM treatment.

There are several reports suggesting that TAM may have synergistic effect when used in combination with different chemotherapeutic agents, including doxorubicin (De Vincenzo et al, 1996), taxanes (Ferlini et al, 1997) and cisplatin (McClay et al, 1993). Although it has been proposed that this chemosensitizing activity of TAM may be due to an inhibition of P-glycoprotein function (Kirk et al, 1993; Leonessa et al, 1994), our group (De Vincenzo et al, 1996) and others (Kang et al, 1993) previously failed to demonstrate this interaction in multidrug resistant (MDR)-bearing human cancer cells. Moreover, TAM potentiates the anti-tumour effects of taxanes also in MDR-negative cancer cell lines (Ferlini et al, 1997). As far as cisplatin is concerned, none of the currently known mechanisms of resistance seem to be influenced by TAM (McClay et al, 1996). Massive apoptosis in our ER-negative models is present at TAM concentrations probably not obtainable in vivo (10 µM). In contrast, undoubtedly oxidative stress has been observed at lower concentrations (< 1 µM), surely obtainable in patients. This ability of TAM to influence cellular redox metabolism may contribute, at least in part, to the positive interaction between TAM and chemotherapeutics: (i) the addition of TAM to doxorubicin, a potent pro-oxidative agent, produces the same effect observed at higher doses of the anthracyclinic drug (De Vincenzo et al, 1995); (ii) the interaction of TAM with cisplatin may be due to the fact that oxidative stress induced by TAM exhausts intracellular thiol levels including glutathione, thereby decreasing cellular detoxifying ability and resistance to platinum compounds (Gosland et al, 1996); (iii) the entry into the cell cycle induced by TAM may sensitize cells to the block in M-phase generated by taxanes. Further experimental work is now needed to test this hypothesis.

In summary, TAM is able to induce oxidative stress, NF-κB modulation and apoptosis of ER-negative human cancer cells. The knowledge of these properties of TAM will contribute to a better understanding of the complex pharmacology of this molecule and consequently to outline the rationale for new clinical protocols in which TAM could be used to maximize the effects of chemotherapy.

### ACKNOWLEDGEMENTS

We thank A Stoler for critical reading of the manuscript and L Molinari for the secretarial assistance. We are also indebted to R Vitalone for the expert technical work.

### REFERENCES

Bass DA, Parce JW, Dechatelet LR, Szejda P, Seeds MC and Thomas M (1983) Flow cytometric studies of oxidative product formation by neutrophils: a graded response to membrane stimulation. *J Immunol* **130**: 1910–1917

*© Cancer Research Campaign 1999*

Berman E, Adams M, Duigou-Ostendorf R, Godfrey L, Clarkson B and Andreef M (1991) Effect of tamoxifen on cell lines displaying the multidrug-resistant phenotype. *Blood* **77**: 818–825

Couldwell WT, Hinton DR, He S, Chen TC, Sebat I, Weiss MH and Law RE (1994) Protein kinase C inhibitors induce apoptosis in human malignant glioma cell lines. *FEBS Lett* **345**: 43–46

De Vincenzo R, Scambia G, Benedetti Panici P, Fattorossi A, Bonanno G, Ferlini C, Isola G, Pernisco S and Mancuso S (1996) Modulatory effect of tamoxifen and ICI 182,780 on adriamycin resistance in MCF-7 human breast cancer cells. *Int J Cancer* **68**: 340–348

Ferlini C, Biselli R, Scambia G and Fattorossi A (1996) Probing chromatin structure in the early phases of apoptosis. *Cell Prolif* **29**: 427–436

Ferlini C, Scambia G, Distefano M, Bombardelli E, Riva A, Fattorossi A, Filippini P, Isola G, Benedetti Panici P and Mancuso S (1997) Synergistic antiproliferative activity of tamoxifen and docetaxel on three oestrogen receptor negative cancer cell lines is mediated by the induction of apoptosis. *Br J Cancer* **75**: 884–891

Gosland M, Lum B, Schimmelpfennig J, Baker J and Doukas M (1996) Insights into mechanisms of cisplatin resistance and potential for its clinical reversal. *Pharmacotherapy* **16**: 16–39

Gundimeda U, Chen ZH and Gopalakrishna R (1996) Tamoxifen modulates protein kinase C via oxidative stress in estrogen receptor-negative breast cancer cells. *J Biol Chem* **271**: 13504–13514

Haldar S, Jena N and Croce CM (1995) Inactivation of Bcl-2 by phosphorylation. *Proc Natl Acad Sci USA* **92**: 4507–4511

Haldar S, Chintapalli J and Croce CM (1996) Taxol induces bcl-2 phosphorylation and death of prostate cancer cells. *Cancer Res* **56**: 1253–1255

Hockenbery DM, Oltvai ZN, Xiao-Ming Y, Milliman CL and Korsmeyer SJ (1993) Bcl-2 functions in an antioxidant pathway to prevent apoptosis. *Cell* **75**: 241–251

Issandou M, Faucher C, Bayard F and Darbon JM (1990) Opposite effects of tamoxifen on in vitro protein kinase C activity and endogenous protein phosphorylation in intact MCF-7 cells. *Cancer Res* **50**: 5845–5850

Jaiyesimi IA, Buzdar AU, Decker DA and Hortobagyi GN (1995) Use of tamoxifen for breast cancer: twenty-eight years later. *J Clin Oncol* **13**: 513–529

Janssen YM, Barchowsky A, Treadwell M, Driscoll KE and Mossman BT (1995) Asbestos induces nuclear factor kappa B (NF-kappa B) DNA-binding activity and NF-kappa B-dependent gene expression in tracheal epithelial cells. *Proc Natl Acad Sci* **92**: 8458–8462

Kang Y and Perry RR (1993) Modulatory effects of tamoxifen and recombinant human α-interferon on doxorubicin resistance. *Cancer Res* **53**: 3040–3045

Kang Y, Cortina R and Perry RR (1996) Role of c-myc in tamoxifen-induced apoptosis in estrogen-independent breast cancer cells. *J Natl Cancer Inst* **88**: 279–284

Kirk K, Houlbrook S, Stuart NSA, Stratford IJ, Harris AL and Carmichael J (1993) Differential modulation of doxorubicin to multidrug and intrinsically drug-resistant cell lines by anti-oestrogens and their major metabolites. *Br J Cancer* **67**: 1189–1195

Leonessa F, Jacobson M, Boyle B, Lippman J, McGarvey M and Clarke R (1994) Effect of tamoxifen on the multidrug-resistant phenotype in human breast cancer cells: isobologram, drug accumulation and Mr 170 000 glycoprotein (gp 170) binding studies. *Cancer Res* **54**: 441–447

Lopes MCF, Vale MGP and Caravalho AP (1990) Ca-dependent binding of tamoxifen to calmodulin isolated from bovine brain. *Cancer Res* **50**: 2753–2758

Los M, Droge W, Stricker K, Bauerle PA and Schulze-Osthoff K (1995) Hydrogen peroxide as a potent activator of T lymphocyte functions. *Eur J Immunol* **25**: 159–165

McClay EF, Albright KD, Jones JA, Christen RD and Howell SB (1993) Tamoxifen modulation of cisplatin sensitivity in human malignant melanoma cells. *Cancer Res* **53**: 1571–1576

McClay EF, Jones JA, Winski PJ, Albright KD, Christen RD and Howell SB (1996) Determinants of tamoxifen sensitivity control the nature of the synergistic interaction between tamoxifen and cisplatin. *Cancer Res* **56**: 3993–3997

Murrell GA, Francis MJ and Bromley L (1990) Modulation of fibroblast proliferation by oxygen free radicals. *Biochem J* **265**: 659–665

Perry RR, Kang Y and Greaves B (1995) Effects of tamoxifen on growth and apoptosis of oestrogen-dependent and -independent human breast cancer cells. *Ann Surg Oncol* **2**: 238–245

Poot M, Kavanagh TJ, Kang HC, Haugland RP and Rabinovitch PS (1991) Flow cytometric analysis of cell-cycle dependent changes in thiol level by combining a new laser dye with Hoechst 33342. *Cytometry* **12**: 184–187

Rao GN and Berk BC (1992) Active oxygen species stimulate vascular smooth muscle cell growth and proto-oncogene expression. *Circ Res* **70**: 593–599

Sen CK and Packer L (1996) Antioxidant and redox regulation of gene transcription. *FASEB J* **10**: 709–720

Wiseman H (1993) Protective actions of tamoxifen and 4-hydroxytamoxifen against oxidative damage to human low-density lipoproteins: a mechanism accounting for the cardioprotective action of tamoxifen? *Biochem J* **292**: 635–638

Wiseman H, Laughton MJ, Arnstein HRV, Cannon M and Halliwell B (1990) The antioxidant action of tamoxifen. Inhibition of lipid peroxidation. *FEBS Lett* **263**: 192–194

Ye Q and Bodell WJ (1996) Production of 8-hydroxy-2′-deoxyguanosine in DNA by microsomal activation of tamoxifen and 4-hydroxytamoxifen. *Carcinogenesis* **17**: 1747–1750

*© Cancer Research Campaign 1999*
